# Ultrasound in primary care: Consensus recommendations on its applications and training. Results of a 3-round Delphi study

**DOI:** 10.1080/13814788.2022.2150163

**Published:** 2022-12-12

**Authors:** Laura Conangla-Ferrin, Pere Guirado-Vila, Mònica Solanes-Cabús, David Teixidó-Gimeno, Lorena Díez-García, Jesus Pujol-Salud, Lidia Evangelista-Robleda, Josefa Bertran-Culla, Yolanda Ortega-Vila, Vicenç Canal-Casals, Antoni Sisó-Almirall

**Affiliations:** Catalan Society of Family and Community Medicine (CAMFiC), EcoAP Network, Barcelona, Spain

**Keywords:** Family medicine, point of care ultrasound, education, quality of care, Delphi study, consensus recommendations

## Abstract

**Background:**

The introduction of portable and pocket ultrasound scanners has potentiated the use of ultrasound in primary care, whose many applications have been studied, analyzed and collected in the literature. However, its use is heterogeneous in Europe and there is a lack of guidelines on the necessary training and skills.

**Objectives:**

To identify the fundamental applications and indications of ultrasound for family physicians, the necessary knowledge and skills, and the definition of a framework of academic and pragmatic training for the development of these competencies.

**Methods:**

A modified 3-round Delphi study was carried out in Catalonia, with the participation of 65 family physicians experts in ultrasound. The study was carried out over six months (from September 2020 to February 2021). The indications of ultrasound for family physicians were agreed (the > = 75th percentile was considered) and prioritised, as was the necessary training plan.

**Results:**

The ultrasound applications in primary care were classified into seven main categories. For each application, the main indications (according to reason for consultation) in primary care were specified. A progressive training plan was developed, characterised by five levels of competence: A (principles of ultrasound and management of ultrasound scanners); B (basic normal ultrasound anatomy); C (advanced normal ultrasound anatomy); D (pathologic ultrasound, description of pathological images and diagnostic orientation); E (practical skills under conditions of routine clinical practice).

**Conclusion:**

Training family physicians in ultrasound may consider seven main applications and indications. The proposed training plan establishes five different levels of competencies until skill in real clinical practice is achieved.

KEY MESSAGES

- The list of ultrasound applications in primary care may be widely extensive.
- Ultrasound training for family physicians should cover clinical knowledge, practical skills in the use of ultrasound scanners and practice options.
- This paper establishes a framework of applications, skills and a training point-of-care ultrasound plan.

## Introduction

The development in recent decades of portable and pocket ultrasound scanners has allowed the point-of-care application of ultrasound, both in physicians’ offices and in the home [1], making it an accessible tool for family physicians.

A systematic review of the applications of point-of-care ultrasound in primary care, published in January 2019 [2], concluded that the precision with which family physicians perform ultrasounds was good in quality studies but also emphasised the importance of training and the disparity of criteria on this point, with wide variations in the duration and type of training received.

There are wide variations in the use of point-of-care ultrasound between European countries [3]. The expansion and introduction of ultrasound into the regular practice of family physicians is already a reality in some European countries while in others, it is still a project in development or an isolated, one-off event.

The ultrasound performed by the family physician should be contextualised in a clinical setting, accompanied by a history and physical examination, and often aimed at answering a specific clinical question [4]. Given the broad spectrum of competencies and tasks performed by family physicians, the ultrasound applications are multiple and varied. Its claimed benefits are limited not only to completing a diagnosis but may also allow risk stratification, ruling out other diagnoses or associated complications, guiding therapeutic decisions, monitoring the response to treatment, serving as a guide for punctures, and screening for some diseases [5].

At the same time, the provision of ultrasound scanners in primary care should be accompanied by thorough, accredited training of professionals. Some specialty programmes include the training of the resident family physician in ultrasound-related competencies, especially in U.S. teaching units [6,7]. However, ultrasound training should be available not only for residents but also for senior specialists. Training may lead to a change in clinical practice and a more remarkable ability to solve ongoing health problems [8].

This study aimed to identify the fundamental applications of ultrasound for family physicians, the necessary knowledge and skills, and the definition of a framework of academic and pragmatic training for developing these competencies.

## Methods

This study was conducted in Catalonia, Spain from September 2020 to February 2021. A literature review was carried out, and the ultrasound applications in primary care were listed [2,4]. Through a modified (includes a joint committee between first and second rounds) 3-round Delphi study, the indications of ultrasound for the family physician and the training plan were agreed and prioritised. All family physicians participating in the Delphi study were members of the ultrasound group of Catalan Society of Family and Community Medicine. Procedure and rounds:

1. Exploratory round: 65 Spanish family physicians trained in clinical ultrasound who routinely use it in their primary care centre. Objective: select the applications considered fundamental from the list obtained from the literature review.
2. First round (core group): 15 family physicians (all belonging to the first group of 65) with accredited experience in ultrasound of more than five years from different primary care centres and with sustained use of ultrasound in the office. Objective: classification of applications and discussion about training requirements.
3. Second round (core group): agreement on the final list of applications and training process. Objective: establish the final document.

**Figure 1.**
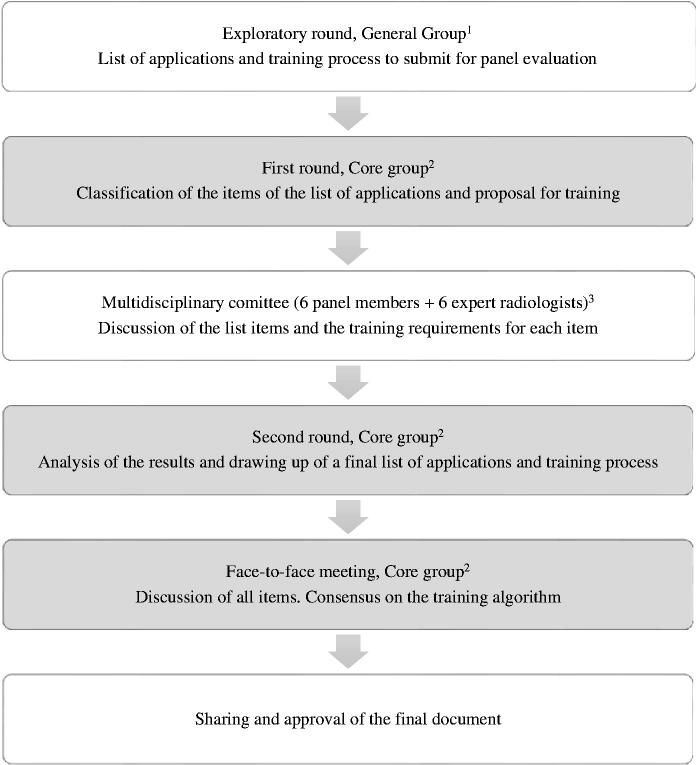
Process flowchart. ^1^General Group, 65 family physicians from the ultrasound group of the Catalan Society of Family and Community Medicine who daily perform ultrasound in their clinical practice; ^2^Core group, 15 family physicians expert in the use of ultrasound, also belonging to the exploratory round group; ^3^6 members of the core group and 6 radiologists who daily perform ultrasound.

Between the first and second rounds, results were discussed in a joint committee composed of six family physicians and six radiologists. Figure 1 shows the diagram followed in the process.

For the development of the training plan, international recommendations from various specialties on the evaluation of competencies in ultrasound [9] were taken into consideration. The competencies agreed in the Delphi study were: (1) indication of ultrasound, (2) knowledge of the ultrasound scanners, (3) image optimisation, (4) systematic examination, (5) image interpretation, (6) ultrasound recording and documentation, and (7) clinical decision making. According to these competencies, a training plan was designed by seeking consensus on the number of hours needed to acquire these skills on each of the applications. It should be considered that these are the minimum hours necessary to establish the learner’s autonomy and continuous training and consulting programmes that guarantee the quality and safety of the patient are required. When the minimum training hours are completed, an exam may accredit the corresponding level of competence.

The results of the Delphi study were analysed with descriptive statistics using the R program version 3.6.1 for Windows. In each round, the ≥75th percentile was considered to assess consensus.

## Results

### Applications

The Delphi study identified seven main ultrasound applications in primary care (Table 1). For each application, different structures to be evaluated raised consensus (Table 1).

**Table 1.** Main applications of ultrasound in primary care.

Abdomen: includes liver, gallbladder and bile ducts, spleen, pancreas, large abdominal vessels, kidneys, bladder, gynaecological/obstetric, prostate and scrotal study
Neck: includes thyroid and parathyroid, major salivary glands (submaxillary and parotid), upper airway, vascular bundles and cervical ganglion chains
Musculoskeletal: includes joint, tendon, muscle and bursa assessment, specific for one of the main joints in primary care: shoulder, elbow, wrist, hip, knee, ankle
Soft parts: dermis and epidermis, subcutaneous fatty tissue
Cardiac: evaluation of heart rate, significant dilation of cavities, global contractility, ejection fraction, pericardial effusion, detection of severe valve disease
Thorax: includes chest wall, pleural pathology (pneumothorax, pleural effusion …), pulmonary pathology with interstitial or alveolar (peripheral) involvement and diaphragmatic assessment
Vascular: includes large vessels, supra-aortic trunks, limb veins…

Applications of ultrasound that were approved by consensus after the 3 rounds (‘Procedures with ultrasound’) was accepted by consensus.

### Indications

Table 2 shows the agreed main indications of ultrasound in primary care according to reason for consultation. The indications also contemplated the ultrasound use to guide different procedures.

**Table 2.** Indications of ultrasound in Primary Care according to reason of consultation, within the different areas of knowledge and competencies.

	Reason for consultation	Main pathologies to be assessed or discarded
**Abdomen** [10] Frasure et al. 2020. USA [11] Esquerrà et al. 2012. Spain [12] Lindgaard et al. 2017. Denmark [13] Speets et al. 2006. Netherlands [14] Sisó-Almirall et al. 2017. Spain	Abdominal pain	Cholelithiasis and cholecystitis Nephritic lithiasic colic and hydronephrosis screening Pancreatitis Appendicitis Gestation (verification of intrauterine gestation, verification of foetal position before imminent delivery), rule out associated complications (ectopic, multiple, retroplacental haematoma, absence of foetal heartbeat in advanced gestation …) Aortic dissection
	Voiding syndrome and/or haematuria	Nephritic lithiasic colic and hydronephrosis screening Increased prostate volume and post-voiding residue Urinary retention Bladder tumour Urinary tract malformation in patients with repeated urinary tract infections
	Palpable abdominal mass	Hepatomegaly and/or splenomegaly Abdominal aortic aneurysm Ultrasound confirmation of abdominal mass to study and associated complications (adenopathy, abdominal free fluid, liver lesions …)
	Scrotal mass	Varicocele Hydrocele Cystic injury with no warning signs Ultrasound confirmation of solid mass and associated complications
**Neck** [15] Tarrazo et al. 2019. Spain	Goitre or thyroid disturbance	Thyroid hyperplasia Thyroid nodule and ultrasound classification of palpable nodules Study of nodule in salivary gland Adenopathy
**Musculoskeletal** [16] Sánchez et al. 2018. Spain [17] Sánchez et al. 2019. Spain	Joint pain	Tenosynovitis, tendinosis, and calcifications Tendon or muscle rupture Joint effusion Bursitis
**Soft parts** [18] Gottlieb et al. 2020. USA	Nodule or palpable surface mass	Lipoma Adenopathy Abscesses and subcutaneous cysts Abdominal wall hernia
**Vascular** [19] Grau et al. 2013. Spain [20] Needleman et al. 2018. USA	High cardiovascular risk	Determination of carotid intima media thickness Presence of atheroma plaques and impact on carotid flow
	Oedema of the lower extremities	Chronic venous insufficiency Deep vein thrombosis Decompensation of heart failure
**Thorax** [21] Diaz et al. 2019. Spain [22] Conangla et al. 2020. Spain [23] Evangelista et al. 2016. Spain	Dyspnoea Pleuritic pain	Interstitial involvement of different causes (interstitial pneumonia, diffuse interstitial lung disease…), especially heart failure Pneumonic pulmonary condensation Structural heart disease (combine with high cardiovascular risk study) Pleural effusion Pneumothorax
**Emergencies** [24] Stengel et al. 2015. Germany	Shock/cardiac arrest	Multiorgan aetiological study Specific protocols: RUSH^1^, SESAME^2^, CAUSE^3^, FATE^4^
	Polytrauma	Free abdominal and thoracic fluid Specific protocols: FAST^5^, eFAST^6^
	Dyspnoea	Acute respiratory failure Specific protocol: BLUE^7^
**Eco-guided procedures** [17] Sánchez et al. 2019. Spain [25] Huang et al. 2015. China	Need of ultrasound guidance for procedure	Drainage of cysts, abscesses, and bruises Channelling of venous or arterial routes Joint and peri-articular punctures Orotracheal intubation Cricothyroidotomy Thoracentesis and paracentesis Lumbar puncture Suprapubic probing

### Training plan

The Delphi study established five levels of competencies (from A to E), and described the expected skills at each level. Table 3 shows the fundamental structure of the training plan, which is progressive. To accede to each level of competence, the previous levels must have been accredited.

**Table 3.** Progressive plan for training in ultrasound in Primary Care.

Level of competence	Concepts and learning method	Assessment and skills
A	Basic physical principles Basic principles of ultrasound and general notions of using the ultrasound scanner. Both concepts can be studied in a theoretical framework (for example, a virtual environment). It includes management of the ultrasound apparatus, as well as basic concepts of image optimisation (definition and concept of the different ultrasound artefacts and how to interpret or minimise them), and the understanding and management of the different modes of examination (B, M, colour Doppler, pulsed Doppler). Must be accredited by theoretical examination (e.g. multiple choice answer type)	Basic physical principles of ultrasound General use of an ultrasound device, including the different modes and basic buttons
B	Normal ultrasound anatomy The support can be in the format of images or videos and face-to-face training is not necessary. It includes ultrasound indications, patient preparation, image optimisation concepts and scanning systematics, as well as the interpretation of normal anatomy images. It must be accredited by means of a theoretical examination that includes the interpretation of images or videos of normal anatomy (both anatomical schemes and ultrasound images)	Normal anatomy of the exploration area Preparation (of the patient) needed to carry out the ultrasound examination of the area
C	Normal ultrasound anatomy It requires face-to-face training through practices in healthy model or simulation model. Special emphasis on image optimisation, scanning systematics, interpretation of normal images and recording and reporting of ultrasound. It must be accredited by practical exam in healthy model or simulation (it is advisable to define and use a checklist of skills)	Perform a systematic ultrasound examination, identifying the different anatomical structures under normal conditions
D	Pathological ultrasound Support may be in image or video format. Special emphasis on the interpretation of the images, recording and documentation of the ultrasound (report with description of the findings), possible diagnostic orientation and clinical decision according to this diagnostic orientation. It must be accredited by means of a theoretical examination that includes the interpretation of pathological images or videos, or by means of a practical examination with a medical simulation mannequin that permits the pathology to be simulated	Possible pathology that affects the exploration area Description of pathological images
E	Ultrasound in conditions of real clinical practice This requires training in real patients, referred for clinical reasons, establishing a concordance between the learner and the tutor. It may be accredited by demonstrating a good agreement with the tutor (not less than 0.7) by direct practical examination or by presenting a portfolio of ultrasounds performed, in the format of clinical cases with the videos recorded and the written reports	Practical skills under conditions of routine clinical practice

The minimum number of hours required to accredit the training is shown in Table 4.

**Table 4.** Minimum number of hours needed to qualify for accreditation of each level of competence.

	A	B	C	D	E
Abdomen	5	25	50	20	50
Neck		8	10	10	25
Musculoskeletal (one joint)		8	10	10	25
Soft parts		8	10	15	25
Chest (cardiac or pulmonary)		8	10	15	25
Vascular		8	10	10	25

## Discussion

### Main findings

This document includes the consensus recommendations of the ultrasound group of the Catalan Society of Family and Community Medicine, on the use of ultrasound in all areas of care of family physicians. This Delphi study establishes a framework of applications and skills and a training plan, which may serve as a guide for training programmes in ultrasound in primary care.

### Applications

The list of ultrasound applications in primary care may be widely extended. A 2019 Danish study described two main approaches to ultrasound for the family physician; focused clinical ultrasound (aimed at a specific question) or exploratory examination, in a wide range of applications [26]. In a 2020 Danish study, a group of local experts prioritized 30 very varied examinations [27], ranging from determining bladder volume to trochanteric bursitis. However, most ultrasound examinations performed in primary care are abdominal, and this is where more family physicians have received ultrasound training [28], followed by musculoskeletal and head and neck ultrasound. In our document, seven areas of knowledge were defined, as well as using ultrasound for guiding procedures.

### Training plan

For the training plan, although levels of competence are typical for any ultrasound, the hours of training required vary according to the different applications. To the accreditation of competencies, the agreed document for assessing of skills in point-of-care ultrasound can be used as a guide [29]. Levels A and B accredit knowledge, level D both accredits knowledge and assesses the skill needed, while levels C and E specifically accredit skills. Level E requires tutored practice in a clinical environment with real patients. For this, the learner must be supervised in carrying out ultrasound examinations by a tutor with accredited experience in ultrasound, until a pre-established number of scans are reached, with an index of agreement with the tutor of >0.7 [30]. We recommend that the tutor is a family physician and that practice is carried out in real clinical conditions. Training for the most advanced level, which involves real clinical practice, may differ. In some cases, the ideal training environment for some ultrasound skills may be the hospital. At this level of advanced skills, collaboration with other specialities is vital.

### Strengths and limitations

The availability of ultrasound in primary care varies widely in Europe. Current scientific evidence and technological progress makes its dissemination advisable requiring an implementation plan that includes a structured and accredited training plan, which guarantees its safe use. In Catalonia, the recent provision of ultrasound scanners in most primary care centres, involved developing a training plan for family physicians. Even though this plan has been developed by consensus of family physicians with expertise in ultrasound, the tremendous territorial variety makes it difficult to establish a common strategy for the implementation of ultrasound in primary care. The different European countries start from very remote situations requiring a specific individualised approach.

### Implications

Ultrasound training for family physicians should cover clinical knowledge, practical skills in using ultrasound scanners and various learning and practice options, both through live tutoring and with consults at a distance with radiology services.

In addition to the knowledge and skills of the technique itself, physicians performing ultrasounds must know the physical principles and operation of ultrasounds and their clinical indications, as well as have the ability to record images and videos in the medical record, write an ultrasound report and make clinical decisions using the information obtained. The creation of referral and consulting circuits that provide communication channels with other related specialities and especially radiology, is highly recommended.

Training family physicians should be accompanied by a national strategic plan for implementing ultrasound. It is necessary to consider not only the benefits but also the risks of introducting ultrasound in primary care and establish a quality training system.

## Conclusion

Training family physicians in ultrasound may consider seven main applications and indications. The proposed training plan establishes five different levels of competencies until skill in real clinical practice is achieved.
